# Emerging Roles and Mechanism of m6A Methylation in Cardiometabolic Diseases

**DOI:** 10.3390/cells11071101

**Published:** 2022-03-24

**Authors:** Zujie Xu, Binbin Lv, Ying Qin, Bing Zhang

**Affiliations:** Division of Sports Science and Physical Education, Tsinghua University, Beijing 100084, China; xuzj20@mails.tsinghua.edu.cn (Z.X.); lbb20@mails.tsinghua.edu.cn (B.L.); qin-y20@mails.tsinghua.edu.cn (Y.Q.)

**Keywords:** N6-Methyladenosine (m6A), cardiometabolic diseases (CMDs), RNA methylation

## Abstract

Cardiometabolic diseases (CMDs) are currently the leading cause of death and disability worldwide, and their underlying regulatory mechanisms remain largely unknown. N6-methyladenosine (m6A) methylation, the most common and abundant epigenetic modification of eukaryotic mRNA, is regulated by m6A methyltransferase, demethylase, and the m6A binding protein, which affect the transcription, cleavage, translation, and degradation of target mRNA. m6A methylation plays a vital role in the physiological and pathological processes of CMDs. In this review, we summarize the role played by m6A methylation in CMDs, including obesity, hypertension, pulmonary hypertension, ischemic heart disease, myocardial hypertrophy, heart failure, and atherosclerosis. We also describe mechanisms that potentially involve the participation of m6A methylation, such as those driving calcium homeostasis, circadian rhythm, lipid metabolism, autophagy, macrophage response, and inflammation. m6A methylation and its regulators are expected to be targets for the treatment of CMDs.

## 1. Introduction

The morbidity and mortality caused by cardiometabolic diseases (CMDs) have been increasing worldwide. The corresponding increases in medical expenses place an enormous economic burden on patients and the healthcare industry [1]. CMDs are multifactorial diseases resulting mainly from the combined effects of genetic and environmental factors. The study of epigenetics refers to heritable modifications in gene expression that are not caused by nucleotide-sequence alterations such as DNA or RNA methylation, or histone or non-coding RNA modifications [2].

Similar to DNA methylation and histone modification, RNA methylation is a reversible modification that regulates gene expression, and is the most common epigenetic modification in RNA nucleotides. More than 170 different modifications have been reported thus far, including N1-methyladenosine (m1A), 7-methylguanosine (m7G), N6-methyladenosine (m6A), and 5-methylcytosine (m5C), which are widely involved in the different biological behaviors demonstrated by RNA in transcription, shearing, nucleation, translation, and degradation [3,4]. The m6A modification refers to methylation of the nitrogen atom at the sixth position of the RNA adenosine; this is the most common and abundant internal modification in eukaryotic mRNA, accounting for approximately 97.4% of known RNA modifications [5]. m6A is also extensively present in non-coding RNAs, including microRNAs (miRNAs), long non-coding RNAs (lncRNAs), and circular RNAs (circRNAs) [6,7,8]. The m6A modification is installed mainly via methylation catalyzed by methylase, removed by demethylase, and recognized by m6A reader proteins that can recognize m6A-modified RNAs [9]. Abundant m6A methylation occurs in the myocardium, in which m6A methylation regulates RNA metabolism to allow for normal functioning of the cardiovascular system. Abnormal expression of key enzymes involved in m6A methylation can change the levels of m6A methylation, which affects metabolic function and causes disorders of RNA transcription and translation, leading to physiologic dysfunction of the cardiovascular system [10].

m6A methylation has become a popular research field of molecular biology due to its crucial regulatory role in biological processes and the pathogenesis of a variety of diseases. Although recent studies have reported the role of m6A modification in cardiovascular disease, the molecular mechanisms by which m6A regulates CMDs remain largely unknown. In this review, we summarize the mechanisms of m6A RNA methylation and the regulatory factors involved in this process, and discuss the roles of m6A methylation in obesity, hypertension, pulmonary hypertension, ischemic heart disease, myocardial hypertrophy, heart failure, and atherosclerosis. We also summarize the current studies examining the mechanisms driving m6A methylation and their roles in the development of CMDs, calcium homeostasis, circadian rhythm, lipid metabolism, autophagy, macro-phage activity, and inflammatory responses. The findings summarized in this review provide future therapeutic targets for the treatment of CMDs.

## 2. Enzymes Involved in m6A Methylation

**Figure 1:**
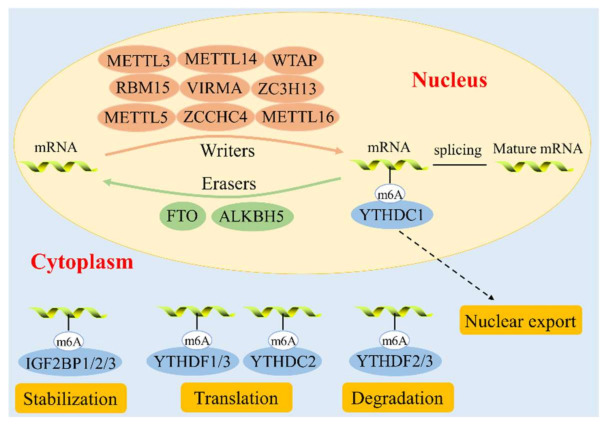
The molecular mechanism of m6A methylation. The methylation of m6A is regulated by m6A methyltransferase, including METTL3, METTL14, WTAP, RBM15, VIRMA, ZC3H13, METTL5, ZCCHC4, and METTL16. FTO and ALKBH5 are demethylases that carry out demethylation. m6A binding proteins, such as YTHDF1/2/3 and YTHDC1/2, recognize specific binding sites to perform a variety of biological functions such as stabilization, translation, and degradation of RNA.

m6A methylation was first discovered in Novikoff hepatic cancer cells in 1974, and was believed at the time to be a broad modification with potential to selectively control gene expression [11]. m6A modification mainly occurs in the 3’-ultranslated region (3’UTR), near stop codons and within internal long exons at the consensus motif RRACH (R = A/G, H = A/C/U) [12,13]. m6A is involved in various stages of RNA metabolism, including stabilization, splicing, nuclear export, translation, and degradation [14]. The dynamic changes in the status of m6A modification and its functions are mainly regulated by protein complexes such as adenosine methyltransferases (writers), demethylating enzymes (erasers), and the m6A-binding proteins (readers) (Figure 1) [15].

### 2.1. m6A Methyltransferase

m6A methylation refers to the process of upregulating the level of RNA methylation using an active methyl group (e.g., S-adenosylmethionine) as the donor under the guidance of a transmethylated protein. This process is mainly catalyzed by the m6A methyltransferase complex (MAC), which includes methyltransferase like (METTL) 3, METTL14, Wilms tumor 1-associated protein (WTAP), RNA-binding motif protein 15 (RBM15), vir like m6A methyltransferase associated (VIRMA), zinc finger CCCH-type containing 13 (ZC3H13), and other components.

The methylase METTL3, which catalyzes the addition of m6A, was first discovered in 1992. METTL3 is an S-adenosyl-L-methionine (SAM) binding subunit that is highly conserved in eukaryotes [16]. As core components of the methyltransferase complex, METTL3 and METTL14 possess similar methyltransferase domains (MTDs), and the two form a heterodimer with a 1:1 co-localization in the nucleus region [17]. As a catalytically active subunit, METTL3 is responsible for transferring a methyl group from SAM or S-adenosylhomocysteine (SAH) to the nitrogen atom in the sixth position of the receptor adenosine [18]. METTL14 binds to an mRNA and assists in the positioning of a methyl group to provide the platform for RNA binding. Thus, METTL14 plays a key role in substrate recognition and enhancing the activity of METTL3 methyltransferase [19]. WTAP is the third type of component in the methylation complex. Because it lacks a highly conserved methylation-catalytic activity center, WTAP does not act as a direct catalyst in methylation. Instead, WTAP recruits the METTL3-METTL14 dimers by using its N-terminal structure to directly bind METTL3. WTAP then delivers the METTL3-METTL14 dimers to the nuclear speckles of nuclear splicing-related organelles, while stabilizing the interaction between METTL3 and METTL14 and acting as a regulatory subunit [20].

New methylation-related enzymes have also been recently discovered. For example, METTL5 combines with the methyltransferase activator TRMT112 to form a heterodimer, which participates in the m6A modification of 18S ribosomal RNA (rRNA). Zinc finger CCHC-type containing 4 (ZCCHC4) is also an m6A modifying enzyme that participates in the modification of m6A at position A4220 of the 28S rRNA [21]. As a methyltransferase, METTL16 not only catalyzes the methylation of U6 snRNA, but also binds to the hairpin structure (hp1) in methionine adenosyl transferase 2A (MAT2A) 3’UTR, thereby regulating the stability and splicing of MAT2A mRNA and SAM homeostasis [22,23]. Our understanding of m6A methyltransferase is still in the exploratory stage. Future functional studies should examine the known components of m6A methyltransferase and screen for the unknown ones.

### 2.2. m6A Demethylase

Demethylation refers to the removal of the m6A modification on the RNA molecule by demethylating the sixth nitrogen atom of adenylate under the guidance of an eraser; this is also the key step in the reversibility of the m6A modification [24]. The main RNA demethylases driving this step are fat mass and obesity-associated protein (FTO) and α-ketoglutarate-dependent homolog 5 (ALKBH5). Although these two enzymes have similar functions, they play different roles in demethylation.

In 2011, FTO, a demethylating protease, was first confirmed in the m6A process, indicating that methylation of N-6 adenine in mRNA is dynamically reversible [25]. FTO sequentially oxidizes m6A into unstable intermediate products N6-hydroxymethyladenosine (hm6A) and N6-formyladenosine (f6A), and then into normal adenosine [26]. The functions of FTO vary depending on whether it is in the nucleus or the cytoplasm. The main nuclear target of FTO is the m6A modification inside mRNA, whereas cytoplasmic FTO mediates demethylation of the m6A modification inside mRNA in addition to the N6,2′-O-dimethyladenosine (m6Am) modification on the 5′ cap of mRNA [27]. Both FTO and ALKBH5 belong to the ALKB family and rely on Fe^2+^ and α-ketoglutarate for their demethylating activity [25,28]. Unlike FTO, ALKBH5 directly removes the methyl group from m6A methylated adenine rather than employing an oxidation step prior to demethylation. The activity of ALKBH5 significantly affects the nuclear export and metabolism of mRNA [28].

### 2.3. m6A Binding Proteins

m6A requires binding proteins in order to recognize specific binding sites. YTH domain-containing family 1/2/3 (YTHDF1/2/3) and YTH domain-containing protein 1/2 (YTHDC1/2) are recognition proteins that specifically recognize the m6A site. YTHDC1 is located in the nucleus and is mainly involved in the transport of mRNA from the nucleus into the cytoplasm [29]. YTHDC1 also participates in the regulation of mRNA splicing [30], while YTHDC2 affects the stability and translation of mRNA by recognizing m6A modifications [31]. YTHDF2, the first protein identified as an m6A reader, accelerates mRNA degradation by selectively recognizing m6A and recruiting the carbon catabolite repression–negative on TATA-less (CCR4-NOT) complex for deadenylation [32]. YTHDF1 and YTHDF3 promote the translation of m6A-modified mRNA [33,34]. The affinity of YTHDF1–3 for m6A methylated mRNA is much higher than that for unmethylated mRNA [35]. There are several other currently known m6A-binding proteins, such as insulin like growth factor 2 mRNA binding protein 1/2/3 (IGF2BP1/2/3), eukaryotic initiation factor 3 (eIF3), and heterogenous nuclear ribonucleoproteins (HNRNP C/G/A2B1), which affect the splicing, translation, stability, and degradation of mRNA.

## 3. Involvement of m6A in CMDs

### 3.1. Obesity

Obesity is an independent risk factor for CMDs [36]. The increased incidence and mortality of various CMDs are closely related to obesity [37]. The World Health Organization officially listed obesity-related cardiomyopathy as a disease in 1948. Obesity cardiomyopathy is characterized as myocardial dysfunction or heart failure in obese individuals independent of other cardiovascular risk factors [38,39].

FTO, a gene closely related to obesity, is an active regulator of energy homeostasis that regulates the m6A levels in human RNA sequences via lipid metabolism [40]. In an independent genome-wide association study, FTO dysfunction was shown to be a high-risk factor leading to early onset and severe obesity in European and Indonesian participants [41]. FTO deficiency in adipocytes leads to an increased body mass that occurs in an m6A-dependent manner. FTO controls body mass by regulating the mobilization of fatty acids in adipocytes [42]. FTO deficiency leads to a reduction in intracellular lipolysis and promotes the storage of fatty acids in adipose tissue [43]. FTO controls exon splicing in adipogenesis regulator Runt-related transcription factor 1 (Runx1t1) by regulating the level of m6A around the splicing site, thereby enhancing the expression of Runx1t1 to regulate mitosis in adipose precursor cells and promote adipogenesis [44,45]. These findings suggest that FTO-mediated demethylation of m6A may be an important target for epigenetic transcription, and it may be instrumental in the regulation of occurrence and development of obesity.

The YTH domain protein is also involved in the occurrence and development of obesity. Single-nucleotide polymorphism of mitochondrial carrier homolog 2 (MTCH2) is associated with obesity and increases fat accumulation in mouse muscle [46]. m6A methylation promotes MTCH2 translation via a YTHDF1-dependent pathway, increases the MTCH2 protein expression level, and promotes adipogenesis [47]. The methyltransferase complex WTAP/METTL3/METTL14 of m6A is also involved in the regulation of adipogenesis. Knockout of this complex leads to cell-cycle arrest and reduces adipogenesis, thereby inhibiting the obesity induced by a high-sugar and high-fat diet in mice [48]. Administration of a high-fat diet for 21 weeks induces upregulation of METTL3 expression, downregulation of FTO expression, and increases in m6A methylation levels in the murine myocardium [49]. Thus, m6A methylation may be a risk factor for obesity cardiomyopathy, although the exact molecular mechanism underlying these events remains unclear.

### 3.2. Hypertension and Pulmonary Hypertension

Hypertension, the main risk factor for cardiovascular and cerebrovascular diseases, results from interactions between genetic and environmental factors. Epigenetics can affect the pathogenesis of hypertension [50]. Using high-throughput sequencing, Wu et al. [51] showed that in the pericytes of the microvessels in spontaneously hypertensive rats, average m6A abundance was reduced but m6A methylation was abundant in the 3’UTR and 5’UTR of mRNA sequences. These results suggest that the pathogenesis of hypertension may be related to a decrease in m6A methylation levels in pericytes.

Pulmonary hypertension is a cardiovascular disease that results in high rates of disability and mortality, yet there is no effective treatment for pulmonary hypertension. Xu et al. [52] showed that pulmonary hypertension may develop and persist because of continuous low expression of the m6A methyltransferase METTL3, which affects the m6A levels of pulmonary hypertension-related genes. Qin et al. [53] indicated that METTL3 mRNA and protein are abnormally upregulated in hypoxia-induced pulmonary hypertensive rats and pulmonary artery smooth muscle cells (PASMCs). Downregulation of METTL3 expression in vitro significantly inhibits the proliferation and migration of PASMCs. Under hypoxic conditions, the levels of the m6A-binding protein, YTHDF2, in PASMCs are significantly increased. YTHDF2 recognizes METTL3-mediated m6A-modified PTEN mRNA and promotes the degradation of PTEN, which in turn activates the PI3K/Akt signaling pathway and leads to excessive PASMC proliferation [53]. In addition to mRNA, circRNAs also contain abundant levels of m6A. Su et al. [54] were the first to reveal the transcriptomic profile of m6A circRNAs in a pulmonary hypertensive rat model. That study demonstrated a significantly reduced abundance of m6A in the circRNAs contained in the lung tissues of rats with hypoxia-induced pulmonary hypertension. In addition, under hypoxic conditions, m6A regulators affects the circRNA-miRNA-mRNA co-expression network, leading to activation of the FoxO and Wnt signaling pathways. The m6A levels of circXpo6 and circTmtc3 are significantly associated with pulmonary hypertension. The above studies suggest that m6A methylation may be an important pathological mechanism in pulmonary hypertension, and that m6A methylation affects circRNAs under hypoxic conditions.

### 3.3. Ischemic Heart Disease

Autophagy is a process used for degradation of intracellular material components via lysosomes in eukaryotic cells. The body uses autophagy to clear senescent organelles and mutant proteins in order to maintain its structural stability and normal function [55]. Autophagy is closely related to ischemic heart disease [56]. Mathiyalagan et al. [57] confirmed for the first time that m6A methylation of mRNA is significantly higher in the ischemic myocardium than in non-ischemic areas. Song et al. [58] showed that the levels of m6A modification and expression of METTL3 are increased in cardiomyocytes under conditions of hypoxia/reoxygenation (H/R) and in the myocardium of mice subjected to ischemia/reperfusion (I/R) injury. No significant changes have been found in the expression of other methylases. That study also showed that METTL3 is the main factor leading to abnormal m6A modification in H/R cardiomyocytes and the myocardium of I/R mice. Overexpression of METTL3 was shown to inhibit autophagic flux and promote apoptosis of H/R cardiomyocytes, suggesting that METTL3 is a negative regulator of autophagy.

Transcription factor EB (TFEB) is a key regulator in the process of lysosomal biosynthesis and autophagy. METTL3 methylates TFEB on two m6A residues of 3’-UTR and promotes the binding of the RNA-binding protein, heterogeneous ribonucleoprotein D (HNRNPD), to the TFEB mRNA precursor, reducing the expression of the TFEB protein [58]. In addition, overexpression of the m6A demethylase ALKBH5 reverses the damaging effects of METTL3 on cardiomyocytes [58]. Despite these findings, it is still unknown whether METTL3 regulates the expression of autophagy-related genes other than that of TFEB, and whether autophagy, in turn, regulates the effects of m6A methylase and demethylase. Cardiac fibrosis, a common pathological change in patients with myocardial infarction, is characterized by abnormal proliferation of cardiac fibroblasts (CFs) and excessive deposition of extracellular matrix (ECM) in the heart. Li et al. [59] showed that silencing METTL3 reduces the levels of m6A modification in fibrosis-related genes, and reduces myocardial fibrosis in mice with myocardial infarction, by inhibiting CF activation. Another study showed that in mice with myocardial infarction, the m6A demethylase ALKBH5 promotes myocardial cell proliferation by demethylating YTHDF1 mRNA and improves heart function [60].

FTO has been shown to exert a positive regulatory effect in mouse models of myocardial infarction. Overexpression of FTO in the myocardial cells of mice subjected to hypoxia or myocardial infarction reduces fibrosis, promotes angiogenesis, and upregulates the expression of myocardial sarcoplasmic/endoplasmic reticulum Ca^2+^ ATPase 2a (SERCA2a), thereby improving the cardiac systolic function [57]. Yang et al. [61] speculated that installing an m6A modification on the lncRNA MALAT1 can be used in the treatment of myocardial ischemia-reperfusion injury. Saxena et al. [62] showed that adjustment of m6A methylation levels improves the efficacy of ischemic myocardial preconditioning, leading to stable expression of myocardial protective proteins and reducing the scope of infarction. The above studies indicate that m6A modification may be a key target in the treatment of ischemic heart disease; however, the specific mechanisms involved require further study.

### 3.4. Cardiac Hypertrophy and Heart Failure

Cardiac hypertrophy is an adaptive response to sustained increase in pressure burden and is characterized by thickening of the ventricular walls. Studies on regulation of gene transcription have shown that post-transcriptional regulation is a key mechanism used to control myocardial hypertrophy [63]. Kmietczyk et al. [64] indicated that the RNA and protein levels of METTL3 in the myocardial tissue of patients with dilated cardiomyopathy are higher than those of healthy individuals, indicating that m6A methylation levels are significantly altered in myocardial hypertrophy.

Myocardial hypertrophy is a complex pathophysiological process involving multiple factors and signaling pathways in cardiomyocytes. Among these signaling pathways, mitogen-activated protein kinase (MAPK) signaling is important in the pathology of myocardial hypertrophy [65]. Dorn et al. [66] showed that the m6A mRNA modification is specifically increased in specific types of proteins after cardiac muscle cells are stimulated using hypertrophic signaling. Overexpression of METTL3 induces remodeling in compensatory cardiac hypertrophy via mitogen-activated protein (MAP)3K6, MAP4K5, and MAPK14, without causing cardiovascular function defects in basal or stressed state. In myocardial-specific METTL3-knockout mice, loss of METTL3 function causes age- and stress-related eccentric cardiomyocyte remodeling and cardiac dysfunction. The findings obtained in this murine model indicate that METTL3 plays an important role in maintaining cardiovascular homeostasis and function, preserving the geometry of cardiomyocytes, and mediating adaptive cardiovascular remodeling after the stress of pressure loading. Studies have shown that piRNAs are involved in pathological processes of myocardial hypertrophy [67]. Gao et al. [68] showed that cardiac-hypertrophy-associated PIWI-interacting RNA (CHAPIR) promotes pathological myocardial hypertrophy and cardiac remodeling by targeting METTL3-mediated m6A methylation of poly (ADP-ribose) polymerase 10 (Parp10) mRNA during transcription. Another study also showed that m6A levels are increased significantly during the development of myocardial hypertrophy, whereas FTO levels in the myocardium are decreased in a time-dependent manner after aortic coarctation. Compared with that of normal mice, the ejection fraction of FTO-knockout mice is significantly decreased after surgery while ventricular dilatation is significantly increased. Additionally, the time from left ventricular hypertrophy to heart failure is significantly shortened in FTO-knockout mice [69]. Moreover, the dysfunctional mutation in human FTO is related to the occurrence of hypertrophic cardiomyopathy, but the mechanisms involved in these events remain undetermined [70].

Heart failure, which is the main cause of death in patients with chronic heart disease, is mainly manifested by decreased heart function and dilatation of the left ventricle. The pathological process of heart failure is accompanied by changes in the levels of cardiomyocyte apoptosis, cardiac fibrosis, and altered gene expression. Hinger et al. [71] showed that, in the myocardial tissue of patients with non-ischemic heart failure, the levels of m6A methylation and METTL3 protein expression are increased, whereas FTO expression is decreased and the levels of ALKBH5 are unchanged, compared with those of healthy myocardial tissues. Zhang et al. [49] indicated that patients and mice with heart failure but preserved ejection fraction (HFpEF) show significant changes in m6A methylation levels. Shen et al. [72] also confirmed that the level of myocardial m6A methylation in heart-failure mice is increased while the expression of FTO is decreased. FTO overexpression reduces H/R-induced cardiomyocyte apoptosis by upregulating the expression of Mhrt. These results suggest that m6A methylation plays an important role in the etiology of heart failure.

### 3.5. Atherosclerosis

Atherosclerosis is a chronic inflammatory disease that is characterized by endothelial dysfunction, monocyte and lymphocyte recruitment, smooth muscle cell migration and proliferation, activation of pro-inflammatory factors, and increased platelet adhesion [73]. Quiles-Jiménez et al. [74] used mass spectrometry to analyze the level of m6A methylation in the tissues of patients with non-atherosclerotic arteries and carotid atherosclerosis, and showed changes in the expression levels of m6A methylase, demethylase, and binding proteins in atherosclerotic tissues. These findings indicate that m6A is involved in the progression of atherosclerosis. Wu et al. [75] showed that the m6A methylation levels in white blood cells of patients and mice with atherosclerosis are significantly reduced. That study also showed that upregulation of ALKBH1 expression is closely related to decreases in the levels of m6A methylation.

Vascular endothelial cell dysfunction is a key factor in the pathogenesis of atherosclerosis. Jian et al. [76] showed that METTL14 expression is increased in endothelial cells stimulated using TNF-α, which promotes the translation of FOXO1 mRNA via YTHDF1 recognition, and increases the expression of adhesion molecules and endothelial-monocyte adhesion. Knockout out of METTL14 significantly inhibits endothelial inflammation and the formation of atherosclerotic plaques, demonstrating the therapeutic potential of METTL14 in the treatment of atherosclerosis. Zhang et al. [77] indicated that METTL14 increases the levels of m6A methylation in pri-miR-19a and promotes the processing of mature miR-19a, thereby promoting the proliferation and invasion of atherosclerotic vascular endothelial cells. The above studies indicate that METTL14-dependent m6A modification can potentially be used as a target in the treatment of atherosclerosis.

## 4. Potential Mechanisms Involved in m6A Methylation-Mediated Regulation of CMDs

### 4.1. m6A Methylation Regulates Calcium Homeostasis

Calcium-ion homeostasis is an important factor in maintaining heart health. SERCA2a, the main subtype of sarcoplasmic reticulum calcium ATPase in cardiomyocytes, plays a major role in the regulation of cardiac contractility [78]. SERCA2a transports cytoplasmic calcium ions into the sarcoplasmic reticulum (SR). The calcium ions stored in the SR are released from the SR with the occurrence of each action potential to induce a myocardial contraction. Myocardial contraction ceases when calcium ions are transported back into the SR [79]. The calcium uptake rate in the SR determines the rate of myocardial relaxation, and the calcium load in the SR determines the amount of calcium ions stored for the next myocardial contraction. Regulation of SERCA2a activity via these two mechanisms controls the contractility and rhythm of the myocardium [80]. Another study showed that mRNA and protein expression of SERCA2a is reduced in mammals with heart failure. Restoring the expression of SERCA2a improves contractile function in cardiomyocytes and prevents the progression of heart failure [81]. Thus, regulation of SERCA2a activity is important in the pathophysiological mechanisms of heart failure.

Mathiyalaganet al. [57] showed that the expression of FTO is downregulated in the cardiomyocytes of mice with heart failure, which causes abnormally increased levels of m6A methylation in the heart and SERCA2a. This cascade leads to abnormal calcium processing and sarcomere dynamics, and ultimately causes loss of cardiomyocyte contractile function. Conversely, overexpression of FTO in stressed hypoxic cardiomyocytes and failed mouse cardiomyocytes alleviates ischemia-induced cardiac remodeling, and loss of cardiac contractile-protein expression and cardiac contractile function; these events manifest as improvement in ejection fraction, short axis shortening rate, and ventricular wall motion in mice at 2 and 4 weeks after myocardial infarction. Comparison of the myocardial m6A profile of single transcripts also showed that protective effects of m6A methylation in cardiomyocytes occur via selective demethylation of SERCA2a by FTO under ischemic conditions. Demethylation of SERCA2a prevents its mRNA degradation, thereby increasing SERCA2a mRNA stability and protein expression [57]. That particular study showed that the levels of m6A methylation are negatively correlated with expression levels of the SERCA2a contractile protein. Thus, we speculate that inhibiting the expression of RNA methyltransferase or overexpressing RNA demethylase may enhance the protein expression of SERCA2a and regulate the stability of calcium ions, thereby improving cardiac systolic function and providing new avenues for the treatment of CMDs. However, whether other m6A regulators can regulate SERCA2a expression and enhance calcium homeostasis remains to be investigated.

### 4.2. m6A Methylation Regulates the Circadian Rhythm

Circadian rhythm is the main influencing factor in CMDs such as hypertension, myocardial infarction, and arrhythmia [82]. Myocardial infarction is more likely to occur in early morning and late night. The infarctions occurring at night result in larger myocardial-infarct areas and higher levels of inflammation in the body, suggesting that circadian rhythm plays a role in the occurrence and severity of myocardial infarction [83]. Circadian-rhythm genes function by inducing mRNA transcription in different organs. m6A methylation affects the translation and degradation of these mRNAs, thereby regulating the circadian rhythm. Fustin et al. [84] showed that m6A methylation modifies a variety of circadian-rhythm genes and affects the rate of circadian rhythm. Specific silencing of METTL3 reduces the level of m6A methylation, thereby disrupting the circadian rhythm and the process of RNA degradation. However, Wang et al. [85] showed that in FTO-knockout mice, circadian rhythm is significantly altered after the methylation level is increased, and overexpression of FTO does not decrease the levels of brain and muscle ARNT-Like 1 (BMAL1). Another study showed that myocardial development is impaired in BMAL1-knockout mice that gradually develop dilated cardiomyopathy [86]. The above studies show that FTO expression levels affect the circadian-rhythm genes. However, future studies need to explore how abnormal expression of FTO affects the expression of circadian-rhythm genes in CMDs.

### 4.3. m6A Methylation Regulates Lipid Metabolism

Disorders of lipid metabolism are involved in the pathogenesis of metabolic diseases such as obesity, diabetes, and hypertension. Zhong et al. [87] showed that fluctuation of mRNA m6A methylation levels in the mouse liver depends on the regulation of a functional circadian clock. Deletion of the mouse liver circadian-rhythm gene BMAL1 leads to the accumulation of reactive oxygen species (ROS), which in turn increases the levels of m6A methylation by increasing the expression of METTL3. In these mice, the mRNA methylation level of PPARα is particularly high, and the accumulation of ROS induces a significant increase in the expression of YTHDF2. Deletion of BMAL1 and the resultant ROS accumulation also affect the transcription and translation of PPARα and downstream lipid metabolism, ultimately leading to increased lipid accumulation. Li et al. [88] showed that liver-specific overexpression of METTL3 aggravates lipid-metabolism disorders and insulin resistance in mice administered a high-fat diet, whereas liver-specific knockout of METTL3 alleviates lipid-metabolism disorders and insulin resistance by extending the mRNA half-life of Lpin1, an important regulator of lipid metabolism. Wu et al. [89] showed that resveratrol reduces the level of liver m6A RNA methylation, upregulates the expression of PPARα, and improves lipid-metabolism disorder in mice administered a high-fat diet. These findings further delineate the relationship between m6A methylation and lipid metabolism. However, future studies need to identify the mechanism of m6A methylation—especially that driving PPARα methylation—in lipid metabolism.

### 4.4. m6A Methylation Regulates Autophagy

Autophagy is an evolutionary-conserved process that degrades dysfunctional proteins and cellular components. It mediates the renewal of organelles and protein degradation, and clears aging or damaged cytoplasmic components [90]. Autophagy homeostasis plays a critical role in the occurrence and progression of CMDs. At basal levels, autophagy protects cardiomyocytes from stress damage, whereas excessive activation of autophagy aggravates myocardial damage and participates in the pathophysiological processes of CMDs [91].

Song et al. [58] investigated the regulatory mechanisms and role of m6A methylation in autophagy in H/R cardiomyocytes. They showed that the level of m6A methylation in the cardiomyocytes of the H/R group is significantly increased, and the level of autophagy is significantly decreased, compared with those of the controls. Silencing METTL3 expression was shown to enhance autophagic flux and inhibit apoptosis in cardiomyocytes of the H/R group. However, overexpression of METTL3 or using small interfering RNA to suppress the expression of the demethylase ALKBH5 was found to inhibit autophagy, promote apoptosis, and aggravate H/R-induced death in cardiomyocytes, suggesting that METTL3 is a negative regulator of autophagy. Moreover, silencing METTL3 increases the autophagic flux by upregulating the expression of the METTL3 downstream target gene, TFEB. Jin et al. [92] showed that FTO-knockout cells contain increased levels of m6A methylation, reduced levels of autophagy marker LC3II, and increased levels of autophagy substrate compared with those of the control group. The expression of FTO significantly increases LC3II expression and consumes autophagy substrates, indicating that FTO positively regulates autophagic processes in an enzymatic activity-dependent manner. In addition, FTO promotes the initiation of autophagy by specifically upregulating the level of Unc-51 like autophagy activating kinase 1 (ULK1). The above studies indicate that m6A methylation plays an important role in the regulation of autophagy. Increased m6A methylation inhibits the occurrence of autophagy and promotes cellular apoptosis, suggesting that m6A methylation regulates the level of autophagy and participates in the physiological processes of CMDs. Thus, regulating autophagy via the targeting of m6A methylation may be an effective strategy for the prevention and treatment of CMDs.

### 4.5. m6A Methylation Regulates Macrophages Response and Inflammation

Various chronic inflammatory diseases stimulate the occurrence and development of CMDs [93]. Interferon regulatory factor-1 (IRF1) plays an important role in regulating immunity, inflammation, and apoptosis in atherosclerosis [94]. Guo et al. [95] showed that overexpression of IRF-1 promotes apoptosis of atherosclerotic macrophages and inflammation by upregulating m6A methylation levels on circ_0029589 and expression of METTL3. METTL3 reduces the inflammatory response of macrophages induced by lipopolysaccharide by inhibiting the NF-κB pathway [96]. Jian et al. [76] showed that knock out of METTL14 inhibits the formation of atherosclerotic plaques by reducing the inflammatory response in endothelial cells.

STAT1 is a key transcription factor that initiates the signaling cascade leading to the activation of pro-inflammatory macrophages. METTL3 has been shown to directly methylate STAT1 mRNA to improve mRNA stability, thereby upregulating the expression of STAT1 and promoting the polarization of M1 macrophages. The polarization of M1 macrophages, mediated by METTL3-STAT1, may lead to the occurrence and progression of atherosclerosis, obesity-related fat remodeling, abdominal aortic aneurysm, and other diseases, rendering it useful as a potential anti-inflammatory target [97].

## 5. Conclusions and Prospective

**Figure 2:**
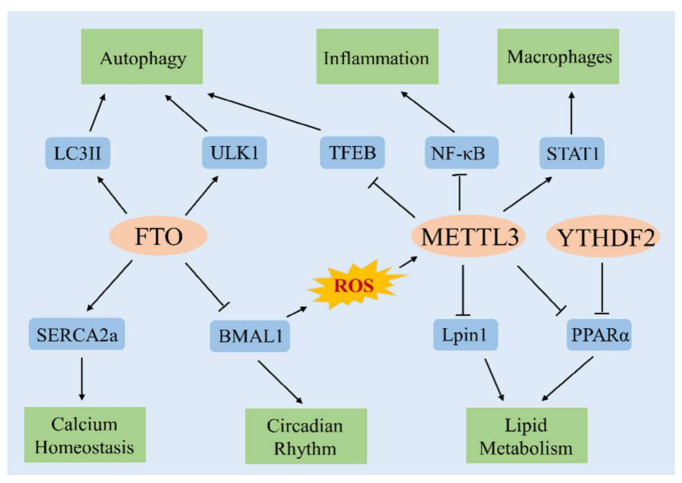
Potential mechanisms of m6A methylation in the regulation of CMDs. FTO regulates calcium-ion homeostasis by increasing the protein expression of SERCA2a, and regulates the circadian rhythm by inhibiting the expression of BMAL1. Deletion of BMAL1 leads to the accumulation of ROS, which in turn regulates the expression of Lpin1 and PPARα by increasing the expression of METTL3. YTHDF2 and METTL3 jointly affect the transcription and translation of PPARα and regulate lipid metabolism. Moreover, silencing of METTL3 upregulates TFEB expression, thereby enhancing autophagy. FTO also promotes autophagy by upregulating the expression of ULK1 and LC3II. METTL3 reduces inflammation by inhibiting the NF-κB pathway and upregulates the expression of STAT1 to promote M1 macrophage polarization. “→” refers to promotion, “—ǀ” refers to suppression.

This review summarizes the pathophysiological effects of m6A methylation in obesity, hypertension, pulmonary hypertension, ischemic heart disease, myocardial hypertrophy, heart failure, atherosclerosis, and other CMDs (Table 1). We also reviewed the potential mechanisms of m6A methylation, including calcium homeostasis, circadian rhythm, lipid metabolism, autophagy, macrophages response, and inflammation, in the regulation of CMDs development (Figure 2).

However, the associations between m6A methylation and CMDs remain to be elucidated. Studies examining m6A methylation in CMDs have mainly focused on METTL3 and FTO expression. Future studies should examine how other methylases and demethylases regulate the expression of downstream proteins under stress, and how the m6A binding proteins mediate the activity and mechanisms of m6A methylation in CMDs. In addition, CMDs are multifactorial diseases, and risk factors, such as smoking, diabetes, nutrition, and aging, should be taken into consideration when examining the pathological mechanisms involved in CMDs [98,99,100]. Current studies show that epigenetic modifications, such as DNA methylation and histone modifications, are related to cardiovascular-risk factors. Nevertheless, it is unclear whether RNA modifications, such as m6A methylation, are also affected by the abovementioned risk factors. In addition, exercise is a non-pharmacological intervention that can alleviate CMDs and directly change the epigenetics of the heart, thereby promoting cardiovascular health and protecting the heart from various pathological processes [101]. However, it is unclear whether exercise changes the phenotype of CMDs by regulating the level of m6A methylation and specifically affecting the downstream targets and signaling pathways of m6A methylase and demethylase. Further exploration of m6A methylation may provide new treatment strategies for CMDs.

## Figures and Tables

**Table 1:** The roles of m6A methylation in CMDs. ——: not mentioned.

Cardiovascular Diseases	m6A-Related Molecules	Expression	Target RNAs	m6A Levels	Main Functions	Reference
Obesity	FTO	Upregulated	Runx1t1	——	Regulates mitosis of fat precursor cells and promotes adipogenesis	[43,44]
	YTHDF1	Upregulated	MTCH2	——	Increases fat accumulation in muscle	[45,46]
	WTAP/METTL3/METTL14 complex	Upregulated	——	Increased	Knockout of this complex causes cell-cycle arrest and reduces adipogenesis	[47]
Pulmonary hypertension	METTL3	Upregulated	——	Increased	Upregulates proliferation and migration of PASMCs	[52]
	——	——	circXpo6 and circTmtc3	Reduced	Possibly affects the circRNA–miRNA–mRNA network	[53]
Ischemic heart disease	METTL3	Upregulated	TFEB	Increased	Inhibits autophagy and promotes apoptosis in cardiomyocytes	[57]
	ALKBH5	Downregulated	YTHDF1	Increased	Promotes the proliferation of cardiomyocytes in mice with myocardial infarction by demethylating YTHDF1 mRNA; improves heart function	[59]
	FTO	Downregulated	SERCA2a	Increased	Overexpression of FTO inhibits myocardial fibrosis and cellular apoptosis, promotes angiogenesis, and improves cardiac systolic function	[56]
Cardiac hypertrophy	METTL3	Upregulated	MAP3K6, MAP4K5 and MAPK14	Increased	Induces remodeling in compensatory cardiac hypertrophy	[65]
	FTO	Downregulated	Mhrt	Increased	FTO overexpression inhibits H/R-induced cardiomyocyte apoptosis	[70,71]
Atherosclerosis	METTL14	Upregulated	miR-19a	Increased	Promotes the proliferation and invasion of vascular endothelial cells	[76]

## Data Availability

Not applicable.
